# The Evidence for Altered BDNF Expression in the Brain of Rats Reared or Housed in Social Isolation: A Systematic Review

**DOI:** 10.3389/fnbeh.2017.00101

**Published:** 2017-05-31

**Authors:** Jana Murínová, Nataša Hlaváčová, Magdaléna Chmelová, Igor Riečanský

**Affiliations:** ^1^Laboratory of Cognitive Neuroscience, Institute of Normal and Pathological Physiology, Slovak Academy of SciencesBratislava, Slovakia; ^2^Laboratory of Pharmacological Neuroendocrinology, Biomedical Research Center, Institute of Experimental Endocrinology, Slovak Academy of SciencesBratislava, Slovakia; ^3^Social, Cognitive and Affective Neuroscience Unit, Department of Basic Psychological Research and Research Methods, Faculty of Psychology, University of ViennaVienna, Austria

**Keywords:** neurotrophic factors, neural plasticity, mental disorders, schizophrenia, depression, animal models, chronic stress, isolation rearing

## Abstract

There is evidence that development and maintenance of neural connections are disrupted in major mental disorders, which indicates that neurotrophic factors could play a critical role in their pathogenesis. Stress is a well-established risk factor for psychopathology and recent research suggests that disrupted signaling via brain-derived neurotrophic factor (BDNF) may be involved in mediating the negative effects of stress on the brain. Social isolation of rats elicits chronic stress and is widely used as an animal model of mental disorders such as schizophrenia and depression. We carried out a systematic search of published studies to review current evidence for an altered expression of BDNF in the brain of rats reared or housed in social isolation. Across all age groups (post-weaning, adolescent, adult), majority of the identified studies (16/21) reported a decreased expression of BDNF in the hippocampus. There are far less published data on BDNF expression in other brain regions. Data are also scarce to assess the behavioral changes as a function of BDNF expression, but the downregulation of BDNF seems to be associated with increased anxiety-like symptoms. The reviewed data generally support the putative involvement of BDNF in the pathogenesis of stress-related mental illness. However, the mechanisms linking chronic social isolation, BDNF expression and the elicited behavioral alterations are currently unknown.

## Introduction

Evidence has been accumulated that development, maturation, and maintenance of neural connections play a critical role in the pathogenesis of major mental illness including depression, schizophrenia, and bipolar disorder. These developmental and homeostatic neural processes are controlled by neurotrophic factors, signaling peptides which act on specific receptors to regulate the physiology of neurons and glial cells (Williams and Umemori, 2014). There are several families of growth factors acting in the brain, including many various molecules. Of these, brain-derived neurotrophic factor (BDNF) has attracted a great deal of attention as probably being importantly involved in various neuropsychiatric diseases (Autry and Monteggia, 2012; Castrén, 2014).

BDNF is a member of the neurotrophin family of growth factors, which also includes nerve growth factor (NGF), neurotrophin 3 (NT-3), and neurotrophin 4 (NT-4; Bothwell, 2014). Similarly to other neurotrophins, BDNF is first synthesized as a precursor protein, proBDNF, which is cleaved to the mature form (Deinhardt and Chao, 2014). BDNF binds primarily to a transmembrane receptor TrkB (tropomyosin receptor kinase B or tyrosine receptor kinase B). NT-4 binds preferentially to TrkB as well, while NGF has highest affinity for TrkA and NT-3 for TrkC receptor. Proneurotrophins, including proBDNF, are also biologically active and all bind to a pan-selective p75 neurotrophin receptor (Lu et al., 2005). Mature neurotrophins are also able to interact with p75 receptor but with low affinity. Through activation of Trk and p75 receptors, respectively, mature neurotrophins and proneurotrophins may produce opposing effects on target cells. Thus, while proBDNF mediates apoptosis and long-term synaptic depression, mature BDNF rather supports neuronal survival, growth, differentiation, and synaptic long-term potentiation (Roux and Barker, 2002; Yoshii and Constantine-Paton, 2010). Throughout the development, BDNF plays a crucial role in cellular proliferation, migration, and phenotypic differentiation (Huang and Reichardt, 2001; Poo, 2001). BDNF is also required in the mature brain for maintenance of neuronal functions, structural integrity of neurons and neurogenesis (Poo, 2001; Autry and Monteggia, 2012).

In patients with schizophrenia, depression and bipolar disorder, reduced expression of BDNF and/or TrkB has been found in the hippocampus and multiple cortical areas (Autry and Monteggia, 2012; Castrén, 2014) and there is also evidence for reduced BDNF levels in the peripheral blood in these disorders (Fernandes et al., 2014). Recent research indicates that BDNF may create an important link between stress and mental illness. Stress is a well-established environmental risk factor of mental diseases and has a potent effect on signaling via BDNF (Gray et al., 2013). Several types of stressors, such as immobilization, foot shocks, or forced swimming have been used to examine BDNF in animal studies and most of them have shown that acute or chronic stress disrupted BDNF signaling in the brain, mainly due to decreases in expression or release (Cirulli et al., 2009; Neto et al., 2011; Bath et al., 2013). In contrast, treatment with antidepressants or antipsychotics was able to prevent, or in some circumstances reverse, the adverse effect of stress on the BDNF pathway (Balaratnasingam and Janca, 2012). In humans, an intensely debated issue is the role of BDNF genotype in susceptibility to neuropsychiatric diseases. A common functional polymorphism in the BDNF gene (termed the Val66Met polymorphism) has been found to interact with stress exposure to affect risk of depression (Hosang et al., 2014), bipolar disorders (Hosang et al., 2010), schizophrenia (Alemany et al., 2011), or suicide (Pregelj et al., 2011).

Social isolation of rats is often used to induce chronic psychosocial stress and study the mechanisms through which psychosocial factors influence pathogenesis of mental and somatic diseases. In humans, the adverse effects of long-term social isolation and psychosocial stress on mental and physical health are well-established (Cacioppo et al., 2011). Rats naturally live in groups and preventing them of social contacts and interaction for a longer time deprives them of important stimuli and represents a significant stressor (Hatch et al., 1965; Hawkley et al., 2012). Chronic social isolation induces a variety of symptoms in rats, including depression-, anxiety-, and psychosis-like behaviors, but also signs of autonomic, neuroendocrine, and metabolic dysregulation (Fone and Porkess, 2008; Karelina and DeVries, 2011; Cacioppo et al., 2015). The terms isolation rearing or isolation housing are commonly used to denote social isolation of adolescent (post-weaning) or adult rats, respectively. The consequences of isolation are more severe in rats compared with other rodent species (Einon et al., 1981) and it has been argued that social isolation of rats has a good etiological validity to model human mental illness (Powell, 2010; Czéh et al., 2016). Given the emerging important role of BDNF in stress-related mental disorders, the aim of our investigation was to systematically summarize current evidence for altered BDNF signaling in experimental studies employing chronic social isolation of rats.

## Materials and methods

### Study identification

We searched Medline PubMed, Scopus, and Web of Science databases for papers and abstracts published until February 2017, written in English, which had investigated BDNF and TrkB in rats exposed to chronic social isolation. Each electronic search was performed by using a specific combinations of key words occurring in title, abstract or paper's key words, such as [(isolat^*^ AND (social^*^ OR reared OR rearing OR housed OR housing) AND (rat OR rats) AND (^*^troph^*^ OR BDNF OR “growth factor^*^”)]. Two researchers (JM and NH) independently screened all titles and abstracts for inclusion. Only primary research reports were considered. Full-text reports of all included references were obtained. We included only studies, which had assessed BDNF and TrkB expression in the brain (at the level of mRNA or protein) of rats isolated for at least 2 weeks. We excluded studies that had employed total isolation, i.e., when the isolated animals had been housed separately so that they had had no visual, auditory, and olfactory contacts with other animals. Studies that had used resocialization following isolation were not excluded. In addition to BDNF and TrkB expression, we also extracted data on the age on isolation onset, isolation duration, strain, sex, behavioral alterations, and other related findings.

## Results

**Figure 1:**
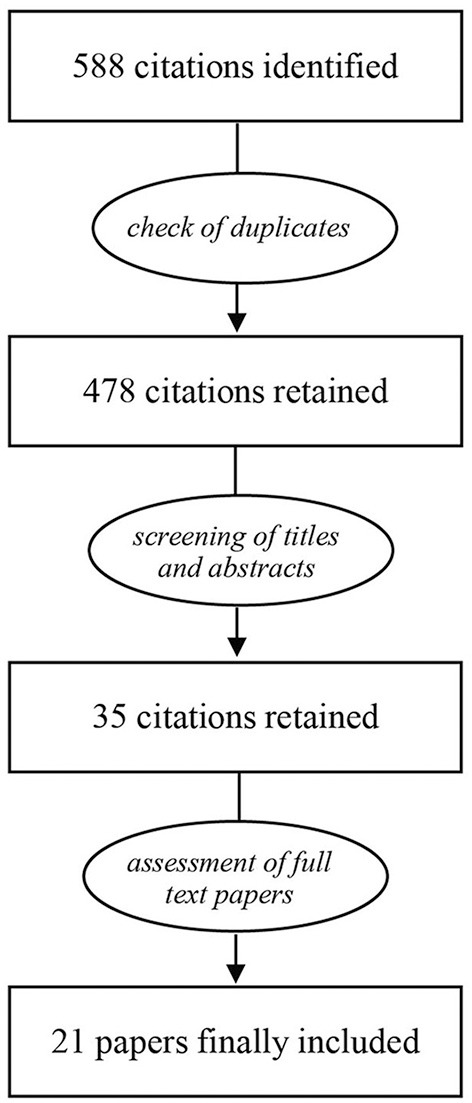
Processing steps that followed the primary search.

The process of selection of papers to be included in the review is shown in Figure 1. After removal of duplicates, the search yielded 478 citations to be screened for inclusion. Following the assessment of titles and abstracts, 35 citations were retained. As a next step, full-text papers were examined, resulting in a final selection of 21 studies. The findings from these studies are summarized in Table 1. Studies were ordered first by the age at the onset of isolation and then by isolation duration (range 2–12 weeks). Most studies (18/21) assessed the expression of BDNF in the hippocampus.

**Table 1:** Summary of results of the studies that employed rearing or housing rats in isolation.

**Isolation onset**	**Isolation duration (weeks)**	**Strain sex**	**Effect on BDNF**	**Additional findings (behavioral, neurobiological, etc.)**	**References**
Post-weaning	4	SD ♀, ♂	= BDNF protein expression in mPFC in both ♂and ♀	↑ Social interaction with a novel conspecific ↑ Aggressive behavior = Locomotion in the home cage = Arc protein expression in mPFC = c-fos protein expression in mPFC = mPFC volume	Wall et al., 2012
Post-weaning	4.3	SD ♂	↓ BDNF protein expression in the hippocampus	↑ Spatial learning (↓ escape latency in MWM) ↓ Despair (↓ immobility in FST) ↑ Anhedonia (↓ sucrose intake) ↓ Arc protein expression in the hippocampus	Pisu et al., 2011a
Post-weaning	4.3	SD ♂	↓ BDNF protein expression in the hippocampus	↑ Anxiety (↓ time in open arms of EPM task) ↓ Arc protein expression in the hippocampus ↓ ALLO levels in the hippocampus	Pisu et al., 2011b
Post-weaning	4.3	SD ♀, ♂	↓ BDNF protein expression in the hippocampus in ♂ = BDNF protein expression in the hippocampus in ♀	↑ Anhedonia in ♂(↓ sucrose intake) ↓ ALLO levels in the cerebral cortex and plasma ↓ Basal corticosterone levels in plasma ↑ Stress-induced corticosterone levels in plasma ↑ CRH protein expression in the hypothalamus in ♂ ↓ CRHR1 protein expression in the pituitary in ♀ ↑ GR protein expression in the hippocampus	Pisu et al., 2016
Post-weaning	5	SD ♂	= BDNF protein levels in the frontal cortex, striatum, hippocampus and cerebellum	= Anxiety (time spent in open arms of EPM) = Spatial learning (escape latency in MWM) = Locomotion (distance in home cage and OF) = NA, DA, 5HT and 5HIAA levels in the frontal cortex, striatum, hippocampus and cerebellum	Simpson et al., 2012
Post-weaning	5.7	L-E ♂	↓ BDNF protein expression in the central amygdala and the hippocampus	= Anxiety (time spent in open arms of EPM) = Basal corticosterone levels in plasma	Ravenelle et al., 2014
Post-weaning	6	SD ♀, ♂	= BDNF protein levels in the frontal cortex, hippocampus, occipital cortex and cerebellar vermis in both ♂and ♀	= NGF protein levels in the frontal cortex, hippocampus, occipital cortex and cerebellar vermis ↑ NT-3 protein levels in the occipital cortex	Parks et al., 2008
Post-weaning	8	SD ♂	↓ BDNF protein level in the striatum	↓ Prepulse inhibition of the startle reflex ↓ Working memory (↓ novel object exploration in NORT)	Uys et al., 2016
Post-weaning	12	F-H ♂	↓ BDNF mRNA expression in the basolateral amygdala and dentate gyrus ↑ BDNF mRNA expression in the retrosplenial cortex	↑ DA D2 receptor binding in NAc, the basolateral amygdala, central nucleus of the amygdala and the substantia nigra ↓ TrkB mRNA expression in the cingulate cortex and the piriform cortex ↑ TrkB mRNA expression in the hippocampus and retrosplenial cortex	Djouma et al., 2006
Post-weaning	2 (+ next 3 weeks of social housing)	SD ♂	↓ BDNF protein expression in the hippocampus and NAc ↑ BDNF protein expression in PFC	Impaired reversal learning in MWM = Spatial learning in MWM	Han et al., 2011
Post-weaning	2 (+ next 3 weeks of social housing)	SD ♂	↓ BDNF mRNA and protein expression in the hippocampus ↑ BDNF mRNA and protein expression in mPFC	↓ H3 acetylation of BDNF gene in the hippocampus ↑ H3 acetylation of BDNF gene in mPFC ↓ Prepulse inhibition of the startle reflex	Li et al., 2016
Post-weaning	2 (+ next 4 weeks of social housing)	SD ♂	↑ BDNF protein expression in PFC and the hippocampus		Meng et al., 2011
Adolescent	6, 10	W ♂	= BDNF protein levels in the hippocampus after 6 weeks of isolation ↓ BDNF protein levels in the hippocampus after 10 weeks of isolation	↑ Anxiety (↑ latency to feed in NSFT) ↑ Despair (↑ immobility in FST) ↓ Hippocampal progenitor cell proliferation and survival ↓ Serum and hippocampal ALLO levels (no differences between the duration of isolation)	Evans et al., 2012
Adolescent	9	W ♂	↓ BDNF protein levels in the hippocampus	= Locomotion (number of line crossing in OF) ↑ Anxiety (↑ latency to feed in NSFT) ↑ Despair (↑ immobility in FST) ↓ Hippocampal progenitor cell proliferation	Sun et al., 2013
Adolescent	2 (+ next 2 weeks of social housing)	W ♂	↑ BDNF protein levels in mPFC = BDNF protein levels in NAc	Deficit in latent inhibition	Shao et al., 2013
Adolescent	2.9 (+ next 2.9 weeks of social housing)	SD ♀, ♂	↓ BDNF mRNA expression in CA3 of the hippocampus in ♀ = BDNF mRNA expression in CA1 and dentate gyrus of the hippocampus	↓ Anxiety (↑ time spent in the open arms of EPM) in ♂ ↑ AVP mRNA expression in PVN in ♀ ↓ Orexin mRNA expression in PVN in ♂ = Basal plasma ACTH levels ↑ Stress-induced plasma corticosterone levels in ♀ ↓ Stress-induced plasma corticosterone levels in ♂	Weintraub et al., 2010
Adulthood	3	W ♂	↓ BDNF mRNA expression in the hippocampus ↑ BDNF mRNA expression in PFC	↓ GR mRNA expression in the hippocampus and PFC ↑ CRH mRNA expression in the hippocampus and PFC ↑ Cdk5 protein levels in PFC ↑ p25 and p35 protein in the hippocampus and PFC ↑ GRS232 phosphorylation in the hippocampus and PFC ↓ JNK1 and JNK 2/3 protein levels in the hippocampus and PFC ↓ Basal serum corticosterone levels	Adzic et al., 2009
Adulthood	5	FRL, FSL ♂	= BDNF mRNA expression in the hippocampus	= Locomotion (distance moved in OF) = Despair (floating in the FST) ↓ Anxiety (↑ time spent in open arm in the EPM) ↓ Working memory (↓ novel object exploration in NORT)	Fischer et al., 2012
Adulthood	6	SD ♂	↓ BDNF protein levels in the hippocampus	↑ Anhedonia (↓ sucrose intake) ↑ Despair (↑ immobility in FST) ↑ Anxiety (↑ latency to feed in NSFT) ↓ DA and 5HT levels in cerebrospinal fluid ↓ Hippocampal progenitor cell proliferation	Ma et al., 2016
Adulthood	8	SD ♂	↓ BDNF expression in the dorsal hippocampus	↓ Synaptophysin expression in the PFC, ventral and dorsal hippocampus, and caudal putamen ↓ PSD93 expression in PFC, ventral and dorsal hippocampus, and amygdala ↓ Number of dendritic spines in PFC, ventral and dorsal hippocampus ↓ pS473-AKT expression in the dorsal hippocampus (active form) ↓ pS9-GSK-3β expression in the dorsal hippocampus (inactive form) ↓ Working memory (↓ novel object exploration in NORT) ↓ Spatial learning (↑ escape latency in MWM task)	Gong et al., 2016
Adulthood	8	SD ♂	↓ BDNF protein levels in the hippocampus = BDNF protein levels in the striatum and PFC	= Basal plasma corticosterone levels	Scaccianoce et al., 2006

*SD, Sprague Dawley; L-E, Long Evans; F-H, Fawn-Hooded; W, Wistar; FRL, Flinders Resistant Line; FSL, Flinders Sensitive Line*.

*♂, male; ♀, female; ↓, decreased; ↑, increased; =, no change*.

*5HIAA, 5-hydroxyindoleacetic acid; 5HT, 5-hydroxytryptamine (serotonin); ACTH, adrenocorticotropic hormone; ALLO, allopregnanolone; Arc, activity-regulated cytoskeleton-associated protein; AVP, arginine vasopressin; BDNF, brain-derived neurotrophic factor; Cdk5, cyclin-dependent kinase 5; CRH, corticotropin releasing hormone; CRHR1, corticotropin releasing hormone type 1 receptor; DA, dopamine; EPM, elevated plus maze; FST, forced swimming test; GR, glucocorticoid receptor; GRS232, glucocorticoid receptor phosphorylation at serine 232; H3, histone protein 3; JNK, c-jun N-terminal kinase; mPFC, medial prefrontal cortex; mRNA, messenger RNA; MWM, Morris water maze; NA, noradrenaline; NAc, nucleus accumbens; NGF, nerve growth factor; NORT, novel object recognition test; NSFT, novelty suppressed feeding test; NT-3, neurotrophin-3; OF, open field; PFC, prefrontal cortex; pS473-AKT, serine-473-phosphorylated Akt (active form); pS9-GSK-3β, serine-9-phosphorylated GSK-3β (inactive form); PSD93, post-synaptic density protein of 93 kDa; PVN, paraventricular nucleus; TrkB, tyrosine receptor kinase B*.

Majority of studies (12/21) employed rats that were reared in isolation from weaning. All 12 studies included the hippocampus in their analyses and nine of them (75%) reported reduced BDNF expression in isolated animals. In one of these studies, decreased BDNF expression was only found in males, while no change was reported in females. Three studies (25%) reported no effect of isolation on BDNF expression in the hippocampus. An increase in BDNF expression in the hippocampus was reported in one study and this study used resocialization of animals following a period of isolation. Six studies analyzed the consequences of post-weaning social isolation on BDNF expression in the frontal cortex. Three of them reported no change, while other three (all using resocialization) found an increase in BDNF expression in isolated animals. Amygdala was investigated in two studies of post-weaning isolation and both reported decreased BDNF expression in isolates. Striatum was analyzed in two studies: one reported a decrease while another one found no change in BDNF expression due to isolation. Overall, no associations between study outcome and duration of isolation were evident.

In four studies, the onset of social isolation was at post-natal day 30–38, i.e., in the adolescent age. Decreased hippocampal BDNF expression in isolated animals was found in three of them (75%). In one of these studies, isolation decreased the expression of BDNF only after sufficiently long isolation (10 weeks) and another study found reduced BDNF specifically in CA3 subregion of the hippocampus and only in female rats. Increased BDNF in the prefrontal cortex (PFC) was found in one study, which used resocialization.

Five studies employed social isolation of adult rats. Four of them (80%) reported decreased BDNF in the hippocampus of isolated animals, while one study found no change. In the prefrontal cortex, one study found no change, while another study reported an increase in the expression of BDNF in isolates.

The vast majority of the studies employed male rats and only four studies also included females. Three of them reported no effect of isolation on BDNF while one study found a decrease specifically in one of the hippocampal subregions. We identified only one study investigating the expression of TrkB receptor. This study reported decreased expression in the cingulate and the retrosplenial cortex but an increase in the piriform cortex and the hippocampus in isolated animals.

The reviewed studies assessed a wide range of behaviors and only few studies assessed the same behavioral domain. Anxiety-like behavior was tested most often (but only in eight studies in total) and was increased following social isolation in 4/8 studies (unchanged in 2/8, decreased in 2/8). Among the studies which found decreased BDNF expression, increased anxiety-like behavior (such as shorter time spent in the open arms of an elevated plus maze or longer latency to feed in the novelty suppressed feeding test) was reported in 4/6 studies (unchanged in 1/6, decreased in 1/6). Among the studies which failed to find decreased BDNF expression, this ratio was 0/2 (unchanged anxiety-like behavior in 1/2, decreased in 1/2). Other symptoms that occurred in studies reporting downregulation of BDNF included increased behavioral despair (indicated by increased immobility in the forced swimming test in 3/4 studies vs. decreased in 1/4), increased anhedonia symptoms (indicated by reduced sucrose preference in 3/3 studies), deficit in working memory (indicated by decreased novel object exploration in the novel object recognition task in 2/2 studies), decreased prepulse inhibition of the acoustic reflex in 2/2 studies (tested only in post-weaning-isolated animals), and impaired spatial learning (as indicated by prolonged escape latencies in the Morris water maze task in 1/3 studies vs. unchanged in 1/3 vs. improved in 1/3).

## Discussion

We systematically reviewed the evidence for altered expression of BDNF and its receptor TrkB in the brain of rats exposed to a long-term (more than 2 weeks) social isolation, which is used to model behavioral and neurobiological phenotype associated with schizophrenia and depression in humans. The identified studies are rather consistent in reporting a decreased expression of BDNF in the hippocampus in isolated animals. This supports the evidence that chronic stress downregulates hippocampal BDNF expression in rats, in line with the findings from other chronic stress paradigms (Duman and Monteggia, 2006; Gray et al., 2013; Numakawa et al., 2013). The decrease in hippocampal BDNF expression was independent of age at the onset of social isolation (i.e., post-weaning period, adolescence, or adulthood). Although there are fewer studies with adolescent and adult animals than with post-weaning-isolated rats, this shows that the effects of stress upon BDNF are not specific for any life period.

Conclusions for other brain regions must be drawn with caution due to lower number of studies compared with those targeting the hippocampus. The current data suggest no significant effects of social isolation on BDNF expression in the cerebral cortex. Future studies should address the cortical (in particular PFC) expression of BDNF more thoroughly since it may be of importance for considering face validity of social isolation as a model for human mental illness. Namely, post-mortem studies in schizophrenia patients quite consistently report decreased BDNF expression in PFC (even more often than in the hippocampus, for review see Reinhart et al., 2015) and thus apparently differ from studies in post-weaning isolation-reared rats, considered as a model for schizophrenia (Powell, 2010; Jones et al., 2011). Interestingly, an elevated BDNF expression in PFC was consistently found in the studies which employed resocialization of animals following a period of isolation. The increased BDNF expression thus might reflect a compensatory mechanism to recover from the detrimental effects of social isolation. The upregulation of BDNF is a well-established effect of antidepressant drugs and is considered to mediate their treatment efficacy (Duman and Monteggia, 2006; Autry and Monteggia, 2012; Castrén, 2014). In contrast to antidepressants which elevate BDNF in both PFC and the hippocampus, resocialization seems not to alter BDNF expression in the hippocampus (the increase was found only in 1/4 studies). Further studies are needed to explore the nature and the physiological significance of this potentially important phenomenon.

It has been observed that chronic stress elevates BDNF and increases growth of dendrites and spines in the amygdala and it was proposed that the differential effects of stress on BDNF and neural plasticity in the hippocampus and the amygdala may play role in the pathogenesis of stress-related mental disorders (Gray et al., 2013; Bennett and Lagopoulos, 2014; McEwen et al., 2016). We identified only two studies targeting the amygdala and both reported decreased BDNF expression. It thus appears that the effects of chronic stress on the amygdala might depend on the experimental paradigm.

We were surprised to identify only one study that had explored the effect of social isolation on the expression of TrkB receptors (Djouma et al., 2006). This study found a decrease in TrkB expression in the cingulate cortex and the piriform cortex but an increase in the hippocampus and the retrosplenial cortex. Given the lack of other reports we can make no consensual statement concerning TrkB expression and further studies are needed to establish more firmly that social isolation affects the expression of TrkB receptors in the brain.

Which are the mechanisms mediating the effects of social isolation on BDNF, in particular the decreased expression in the hippocampus? Surprisingly, among all the studies included in this review, only two addressed the possible mechanisms toward reduced hippocampal BDNF expression. Li et al. (2016) reported that post-weaning isolation rearing had decreased BDNF mRNA and protein expression in the hippocampus, which was accompanied with decreased acetylation of histone H3 of the BDNF gene, suggesting that histone modifications could play a role in downregulating BDNF. However, it is not clear that these changes were due to social isolation since this study used resocialization of rats after isolation. The study by Adzic et al. (2009) revealed modifications of glucocorticoid receptors following isolation of adult rats. The authors argued that these changes could decrease the transcriptional activity of the glucocorticoid receptor upon the BDNF gene. Such a lack of empirical data does not allow us to draw any conclusions on the mechanisms underlying the reduced hippocampal BDNF expression in socially isolated rats. Since the discussion of the engaged processes remains hypothetical so far, we will just briefly address the major candidate mechanism here.

The fact that isolation negatively affects both BDNF protein and mRNA levels in the hippocampus (see Table 1) indicates a reduced gene transcription rather than involvement of post-transcriptional processes. The transcription of the BDNF gene is regulated by transcription factors and epigenetic chromatin modifications (West et al., 2014). The BDNF gene of rodents (and also humans) is complex, containing several exons linked to separate promoters which interact with multiple transcription factors, including, among others, cAMP/Ca^2+^-response element binding protein (CREB), activator protein-1 (AP-1), and nuclear factor kappa B (NF-κB). Epigenetic regulation of the BDNF transcription is achieved by modifications of DNA (methylation) and histone proteins (methylation, acetylation) and also involves the action of methylated CpG-binding proteins, such as MePC2 (Boulle et al., 2012). Both transcription factors and chromatin modifications have been implicated in stress-related changes of BDNF gene transcription.

It has been suggested that the reduction of BDNF in chronic stress is due to a long-term exposure to high levels of adrenal glucocorticoids (GCs, predominantly corticosterone in rats), which are the major mediator of the stress response (Gray et al., 2013; Numakawa et al., 2013; Suri and Vaidya, 2013; Castrén, 2014). This argument is based largely on the well-established finding that chronic treatment with high doses of CGs downregulates BDNF expression in the hippocampus (Chao and McEwen, 1994; Smith et al., 1995; Dwivedi et al., 2006; Jacobsen and Mørk, 2006). While the existence of glucocorticoid response elements within the BDNF gene is probable (Hansson et al., 2006), GCs may affect BDNF gene transcription also by their interactions with other transcription factors, such as CREB or AP-1 (Schaaf et al., 2000; Suri and Vaidya, 2013; Castrén, 2014) and may also act via epigenetic modifications of the BDNF gene (Stankiewicz et al., 2013). However, several findings are not consistent with the idea that GCs play a central role in the reduced transcription of the BDNF gene in chronic social isolation. First, there is little evidence for a chronic increase in basal GC levels in socially isolated rats (Weiss et al., 2004; Serra et al., 2005; Fone and Porkess, 2008; Cacioppo et al., 2015). Also the studies included in this review reported unchanged or decreased basal plasma corticosterone concentrations. Though, it is possible that basal GC concentrations are only increased during an early phase of isolation, which may be sufficient to induce lasting changes of BDNF transcription. Second, the inhibition of BDNF expression by stress exposure occurs also in adrenalectomized rats, in which the physiological stress-related elevations of corticosterone are eliminated (Smith et al., 1995). Finally, Li et al. (2017) have recently demonstrated that the effects of a prolonged corticosterone administration differ between adolescent and adult rats: Hippocampal expression of BDNF was decreased in the adult, but increased in the adolescent rats. Social isolation, in contrast, seems to decrease hippocampal BDNF irrespective of age at which the animals are isolated. These findings indicate that the downregulation of BDNF after chronic isolation cannot be attributed in a simple and straightforward manner to high levels of GCs. Research shows that the relationship between GCs and BDNF is complex and should be rather viewed as complementary, whereby GCs and BDNF jointly contribute to stress adaptation (Gray et al., 2013; Jeanneteau and Chao, 2013; Numakawa et al., 2013; Suri and Vaidya, 2013; Castrén, 2014).

Numerous findings show a link between BDNF and the serotoninergic system (Martinowich and Lu, 2008; Homberg et al., 2014). Stress, including social isolation stress, exerts multiple influence on the serotoninergic system (Chaouloff et al., 1999; Weiss and Feldon, 2001; Fone and Porkess, 2008; Mahar et al., 2014). Serotonin signaling has an influence on BDNF gene transcription through altered activity of CREB, but also other transcription factors (Martinowich and Lu, 2008; Homberg et al., 2014). Moreover, there is evidence that serotonin can affect BDNF chromatin remodeling (Ignácio et al., 2014). This suggest that the serotoninergic system might play a role in mediating the effects of social isolation on BDNF expression. There is also increasing evidence that chronic social isolation impairs antioxidant defenses and disrupts redox homeostasis in the brain (Schiavone et al., 2009; Filipović et al., 2017). BDNF transcription factors NF-κB and AP-1 are sensitive to oxidative and nitrosative status (Kamata et al., 2002; Parohova et al., 2009) and have been implicated to play a role in the pathogenesis of neuropsychiatric disorders, including schizophrenia and depression (Altinoz et al., 2016; Ménard et al., 2016). Yet relatively little is known about this potentially important pathway linking stress and BDNF expression in the brain.

There are few doubts that social isolation is stressful for rats (Hatch et al., 1963). Besides stress, however, another consequence of isolation is an overall decrease in sensory input and motor activities. It is known that sensory stimulation and physical activity both stimulate BDNF expression (Cotman et al., 2007; Karpova et al., 2010; Phillips et al., 2014; Sale et al., 2014). Therefore, in the search for mechanistic links toward the decreased BDNF expression in social isolation of rats, sensory-motor deprivation should also be considered as a possible causal factor.

Since the methods of behavior assessment varied considerably among the studies, to summarize the results across the studies was uneasy. Anxiety-like behavior was the only domain where the occurrence of the symptoms could be compared between the studies which had found or had not found the decreased BDNF expression. Increased anxiety-like behavior was present more often when BDNF expression in the brain was decreased, suggesting a link between BDNF and anxiety symptoms. Depression-like symptoms (the signs of behavioral despair or anhedonia), deficits in working memory and sensorimotor gating also co-occurred with decreased BDNF expression. However, data are too scarce to enable us to analyze the behavioral changes as a function of BDNF expression. Moreover, it remains to be established whether the decreased BDNF expression plays a causal role in the pathogenesis of the isolation-induced behavioral alterations (cf. Taliaz et al., 2010). Surprisingly, none of the reviewed studies analyzed the pathways downstream of BDNF/TrkB such as the phospholipase C pathway, the PI3K/Akt (phosphatidylinositol 3-kinase/protein kinase B) pathway, or the MAPK/ERK (mitogen-activated protein kinase/extracellular signal related kinase) pathway, which mediate the effects of BDNF on synaptic plasticity and neurogenesis in the hippocampus (Ninan, 2014). For instance, recent evidence indicates that mTORC1 (mechanistic target of rapamycin complex 1), a target of MAPK/ERK signaling, is inhibited by chronic stress and is involved in stress-induced synaptic dysfunction and depression-like behavioral changes (Ota et al., 2014; Duman et al., 2016, see also Zhou et al., 2014). Due to decreased BDNF, the activity of the MAPK/ERK/mTORC1 pathway might be compromised in chronic social isolation. Yet, the involvement of this as well as other BDNF-sensitive candidate mechanisms awaits empirical exploration.

To conclude, there is a good evidence that the expression of BDNF is decreased in the hippocampus of rats reared or housed in social isolation. Current data do not indicate that the expression of BDNF is changed in the cerebral cortex. There are too few data to draw conclusions for other brain regions. The expression of BDNF seems to be increased in PFC in animals that were returned to social housing after a prolonged period of isolation, indicating a compensatory mechanism. Studies are lacking on the effects of social isolation on the expression of TrkB receptors in the brain. In general, the research findings are in agreement with putative involvement of BDNF in the pathogenesis of stress-related mental disorders. However, almost entirely absent are empirical data on the neurobiological mechanisms underlying the altered BDNF expression as well as the involvement of signaling pathways downstream of BDNF in chronically isolated animals. Given the established use of social isolation to model the symptoms of schizophrenia and depression, future research should focus on exploring the mechanistic links between chronic social isolation, BDNF expression and the elicited behavioral alterations.

## Author contributions

JM and NH performed the literature search and assessed full-text papers for inclusion. MC and NH extracted the data. JM, MC, NH, and IR wrote the manuscript.

### Conflict of interest statement

The authors declare that the research was conducted in the absence of any commercial or financial relationships that could be construed as a potential conflict of interest.
